# Deep Learning Improves Robustness of Voxelwise Kinetic Modeling for Hyperpolarized Carbon‐13 MRI

**DOI:** 10.1002/mrm.70549

**Published:** 2026-08-04

**Authors:** Kofi Deh, Yeona Kang, Tsang‐Wei Tu, Rania Jones, Curtiland Deville, Rao Khan

**Affiliations:** ^1^ Department of Physics and Astronomy Howard University Washington DC USA; ^2^ Department of Mathematics Howard University Washington DC USA; ^3^ College of Medicine Howard University Washington DC USA; ^4^ Department of Radiation Oncology Johns Hopkins Medicine Washington DC USA

**Keywords:** hyperpolarized carbon‐13 MRI, kinetic modeling, neural networks

## Abstract

**Purpose:**

To evaluate whether deep learning improves the robustness of voxelwise kinetic parameter estimation from hyperpolarized (HP) ^13^C MRI compared with nonlinear least‐squares (NLLS) fitting.

**Methods:**

A hybrid neural network (NN) was trained on synthetic pyruvate/lactate time courses generated from an open‐system two‐compartment HP ^13^C signal model to estimate the pyruvate‐to‐lactate conversion rate ($k_{\mathrm{PL}}$), vascular‐extravascular exchange rate ($k_{\mathrm{VE}}$), and vascular volume fraction ($v_{B}$). NN performance was compared with NLLS across flip‐angle schemes, SNR levels, perturbations in acquisition parameters, and in vivo. Matched‐ratio simulations tested whether model‐estimated ($k_{\mathrm{PL}}$) retained information beyond the Lac/Pyr area‐under‐the‐curve ratio, ${\mathrm{AUC}}_{\mathrm{Lac}/\mathrm{Pyr}}={\mathrm{AUC}}_{\mathrm{Lac}}/{\mathrm{AUC}}_{\mathrm{Pyr}}$.

**Results:**

In simulations, NLLS and NN performance were comparable for $k_{\mathrm{PL}}$ estimation at high SNR, whereas the NN outperformed NLLS at low SNR and for the weakly identifiable parameters $k_{\mathrm{VE}}$ and $v_{B}$. In vivo, NN maps were more spatially coherent than NLLS maps: $k_{\mathrm{PL}}$ corresponded with ${\mathrm{AUC}}_{\mathrm{Lac}/\mathrm{Pyr}}$, while $k_{\mathrm{VE}}$ and $v_{B}$ corresponded with pyruvate AUC. In matched‐ratio simulations, NLLS‐derived $k_{\mathrm{PL}}$ discriminated the metabolic classes better than NN‐derived $k_{\mathrm{PL}}$, although both model‐based estimates retained discriminatory information.

**Conclusion:**

NLLS is effective for $k_{\mathrm{PL}}$ estimation under ideal model‐matched conditions, whereas the NN provides more stable voxelwise maps, especially for weakly identifiable parameters and under low‐SNR or in vivo conditions. Prospective biological or repeatability validation is needed to establish quantitative accuracy.

## 1 Introduction

Hyperpolarized (HP) ^13^C MRI with [1–^13^C]pyruvate enables voxel‐wise quantification of real‐time metabolic conversion in vivo, providing spatiotemporal tissue metabolism for both research and clinical applications [1, 2, 3, 4, 5, 6, 7]. Two readouts are widely used as surrogates of pyruvate‐to‐lactate conversion: (i) the area‐under‐the‐curve ratio, ${\mathrm{AUC}}_{\text{ratio}}={\mathrm{AUC}}_{\text{Lactate}}/{\mathrm{AUC}}_{\text{Pyruvate}}$, and (ii) the apparent pyruvate‐to‐lactate exchange rate, $k_{\mathrm{PL}}$, obtained from voxel‐wise kinetic modeling. ${\mathrm{AUC}}_{\text{ratio}}$ is simple and SNR‐efficient because it aggregates signal over the full time‐course, but it conflates metabolic conversion with delivery, timing, flip‐angle schedule, relaxation, and amplitude‐scaling effects. Common receive sensitivity within a voxel largely cancels in the lactate/pyruvate ratio, but spatial receive inhomogeneity, polarization level, and preprocessing or calibration differences can still affect absolute signal amplitudes and model‐based parameter estimation [8, 9, 10]. In contrast, $k_{\mathrm{PL}}$ offers mechanistic interpretability: it is a rate constant in the governing ordinary differential equations (ODEs) and once flip angle, TR and T1 effects are modeled, it quantifies conversion on an absolute time scale. Joint modeling of pyruvate and lactate further separates variance attributable to vascular fraction $v_{B}$, exchange/delivery $k_{\mathrm{VE}}$, and conversion $k_{\mathrm{PL}}$, which ${\mathrm{AUC}}_{\text{ratio}}$ cannot distinguish. Recent multicenter consensus recommendations further emphasize harmonization of system calibration, acquisition, reconstruction, and quantitative analysis for HP ^13^C pyruvate MRI studies [11].

However, voxelwise estimation of $k_{\mathrm{PL}}$ with conventional two‐compartment nonlinear least‐squares (NLLS) fitting can exhibit substantial variance, particularly at low SNR and under acquisition mismatches (e.g., timing offsets, effective flip angle errors, and scaling differences) [8]. This motivates methods that retain the interpretability of kinetic parameters while approaching the robustness and computational efficiency of AUC ratio‐based analysis.

Deep neural networks (DNNs) have shown promise for denoising and inverse modeling under imperfect measurements [12, 13, 14, 15]. Recent work has also used a spatially constrained U‐Net trained with a digital brain phantom to estimate $k_{\mathrm{PL}}$ maps from dynamic HP ^13^C MRI, further demonstrating the potential of learned regularization for pharmacokinetic mapping [16]. Unlike that spatially constrained approach, the network proposed in this study operates on paired voxelwise temporal signals and jointly estimates $k_{\mathrm{PL}}$, $k_{\mathrm{VE}}$, and $v_{B}$. A key barrier in HP ^13^C MRI is the limited availability of large, standardized in vivo datasets for supervised training. Here we address this gap by (1) training a compact hybrid multi‐head neural network on synthetically generated dynamic pyruvate/lactate data spanning realistic SNRs and protocol perturbations; and (2) demonstrating, in simulation and in vivo studies, that the proposed method reduces sensitivity to delivery‐ and gain‐related confounds and yields physiologically consistent $k_{\mathrm{PL}}$, $k_{\mathrm{VE}}$, and $v_{B}$ maps compared to NLLS, while maintaining consistency with major spatial patterns observed in AUC maps. Absolute biochemical validation remains an open frontier, but the results indicate that physics‐aware DNNs can narrow the gap between simple ratios and fully mechanistic modeling in HP ^13^C MRI. We evaluate the proposed method against voxelwise NLLS fitting in simulation across multiple SNR and acquisition‐perturbation conditions, and in representative preclinical and clinical HP ^13^C datasets.

## 2 Methods

### 2.1 Signal Model

We used an effective open‐system two‐compartment signal model for dynamic hyperpolarized ^13^C pyruvate/lactate imaging. Let $P_{v}\left(t\right)$ denote the vascular pyruvate input, $P_{e}\left(t\right)$ the extravascular pyruvate concentration‐equivalent signal, and $L_{e}\left(t\right)$ the extravascular lactate concentration‐equivalent signal (Figure 1). The measured voxel pyruvate signal, $S_{\mathrm{pyr}}\left(t\right)$, was modeled as the volume‐weighted sum of vascular and extravascular pyruvate,

(1) $$S_{\mathrm{pyr}}\left(t\right)=v_{B}P_{v}\left(t\right)+\left(1-v_{B}\right)P_{e}\left(t\right)$$

where $v_{B}$ is the vascular volume fraction. The measured lactate signal, $S_{\mathrm{lac}}\left(t\right)$, was assumed to arise from the extravascular lactate concentration‐equivalent signal,

(2) $$S_{\mathrm{lac}}\left(t\right)=\left(1-v_{B}\right)L_{e}\left(t\right)$$

**FIGURE 1:**
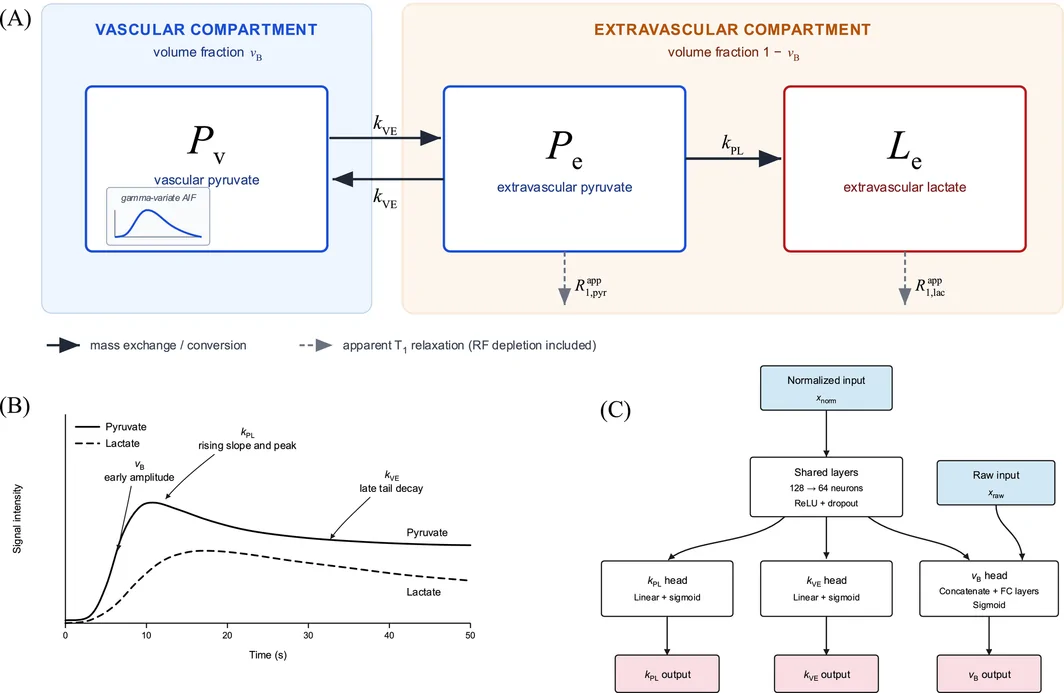
(A) Effective open‐system two‐compartment HP‐^13^C model: The vascular pyruvate input $P_{v}\left(t\right)$ is represented by a gamma‐variate AIF, extravascular pyruvate, $P_{e}\left(t\right)$, is driven by concentration‐dependent vascular–extravascular exchange with apparent rate, $k_{\mathrm{VE}}$, and extravascular pyruvate converts irreversibly to lactate at $k_{\mathrm{PL}}$. Both metabolites experience relaxation and RF‐depletion losses ($R_{1P},\theta_{P}$; $R_{1L},\theta_{L}$). (B) Schematic pyruvate (solid) and lactate (dashed) time courses highlighting parameter influence ($v_{B}$: Early amplitude; $k_{\mathrm{PL}}$: Rise/peak; $k_{\mathrm{VE}}$: Late decay). (C) Hybrid NN: Robust‐normalized input ($x_{\mathrm{norm}}$) passes through shared layers; heads predict $k_{\mathrm{PL}}$ and $k_{\mathrm{VE}}$ (sigmoid → physical range), while $v_{B}$ head concatenates shared features with raw input ($x_{\mathrm{raw}}$).

Radiofrequency (RF) depletion was incorporated into metabolite‐specific apparent longitudinal relaxation rates. For each metabolite M∈{Pyr,Lac}, the apparent relaxation rate at acquisition time $t_{k}$ was

(3) $$R_{1,M}^{\mathrm{app}}\left(t_{k}\right)=R_{1,M}-\frac{\ln\left[\cos\theta_{M}\left(k\right)\right]}{\mathrm{TR}}$$

where $R_{1,M}$ is the intrinsic longitudinal relaxation rate, $\theta_{M}\left(k\right)$ is the flip angle for metabolite M at time point k, and TR is the repetition time. In the forward integration, $R_{1,M}^{\mathrm{app}}\left(t_{k}\right)$ was treated as piecewise constant over each acquisition interval, with $R_{1,M}^{\mathrm{app}}\left(t\right)=R_{1,M}^{\mathrm{app}}\left(t_{k}\right)$ for $t\in \left[t_{k},t_{k+1}\right)$; no temporal interpolation of the flip‐angle schedule was performed. Pyruvate exchange between the vascular and extravascular compartments was modeled as a first‐order process, and pyruvate‐to‐lactate conversion was modeled as irreversible conversion from extravascular pyruvate to extravascular lactate. The governing equations were

(4) $$\frac{dP_{e}\left(t\right)}{\mathrm{dt}}=k_{\mathrm{VE}}P_{v}\left(t\right)-\left[k_{\mathrm{VE}}+k_{\mathrm{PL}}+R_{1,\mathrm{pyr}}^{\mathrm{app}}\left(t\right)\right]P_{e}\left(t\right)$$

(5) $$\frac{dL_{e}\left(t\right)}{\mathrm{dt}}=k_{\mathrm{PL}}P_{e}\left(t\right)-R_{1,\mathrm{lac}}^{\mathrm{app}}\left(t\right)L_{e}\left(t\right)$$

where $k_{\mathrm{VE}}$ is the apparent vascular‐extravascular exchange rate and $k_{\mathrm{PL}}$ is the apparent pyruvate‐to‐lactate conversion rate. For synthetic data generation and gamma‐variate NLLS fitting, the vascular pyruvate input was modeled as

(6) $$P_{v}\left(t;A,\alpha ,\beta ,t_{0}\right)=A\frac{\tau {\left(t\right)}^{\alpha }\exp\left[-\beta \tau \left(t\right)\right]}{\max_{j}\left\{\tau {\left(t_{j}\right)}^{\alpha }\exp\left[-\beta \tau \left(t_{j}\right)\right]\right\}},\tau \left(t\right)=\max\left(t-t_{0},0\right)$$

where A is the peak vascular‐input amplitude, α and β control the bolus shape and decay, $t_{0}$ is the arrival time, and the denominator normalizes the sampled input curve to unit peak before scaling by A. Because $P_{v}\left(t\right)$ is represented as a gamma‐variate vascular input/AIF rather than as a locally mass‐conserved vascular state variable, this formulation should be interpreted as an open input‐driven voxel model; upstream delivery and downstream washout are absorbed into $P_{v}\left(t\right)$, and local closed‐system mass conservation between $P_{v}\left(t\right)$ and $P_{e}\left(t\right)$ is not imposed. Equations ((1), (2), (3), (4), (5), (6)) were integrated forward from initial conditions, $P_{e}\left(t_{0}\right)=0$ and $L_{e}\left(t_{0}\right)=0$, to generate predicted pyruvate and lactate signals.

### 2.2 NLLS Fitting

For nonlinear least‐squares fitting, the vascular pyruvate input $P_{v}\left(t\right)$ was represented by the gamma‐variate function in Equation (6) and fit jointly with the kinetic parameters (see Note S1 for a brief practical‐identifiability assessment of this gamma‐variate NLLS formulation). The fitted parameter vector was $\theta =\left[k_{\mathrm{PL}},k_{\mathrm{VE}},v_{B},A,\alpha ,\beta ,t_{0}\right]$. For each candidate θ, $P_{v}\left(t\right)$ was generated from Equation (6), $P_{e}\left(t\right)$ and $L_{e}\left(t\right)$ were obtained by forward integration of Equations (4) and (5) and predicted pyruvate and lactate signals were computed using Equations (1) and (2). The optimal parameters were obtained by minimizing the weighted residual error across both pyruvate and lactate time courses:

(7) $$\begin{array}{cc} \hat{\theta } & =\arg\min_{\theta }\left[\sum_{i}{\left(\frac{S_{\mathrm{pyr}}^{\text{pred}}\left(t_{i};\theta \right)-S_{\mathrm{pyr}}^{\mathrm{obs}}\left(t_{i}\right)}{\sigma_{\mathrm{pyr}}}\right)}^{2}\right.\\ & \;+\left.\sum_{i}{\left(\frac{S_{\mathrm{lac}}^{\text{pred}}\left(t_{i};\theta \right)-S_{\mathrm{lac}}^{\mathrm{obs}}\left(t_{i}\right)}{\sigma_{\mathrm{lac}}}\right)}^{2}\right] \end{array}$$

Here, $S_{\mathrm{pyr}}^{\mathrm{obs}}$ and $S_{\mathrm{lac}}^{\mathrm{obs}}$ are the observed pyruvate and lactate time courses, and $\sigma_{\mathrm{pyr}}$ and $\sigma_{\mathrm{lac}}$ are fixed channel‐specific residual scaling factors. When independent estimates of the noise standard deviation were available, for example from simulation or background measurements, these values were used directly. When such estimates were unavailable, we used fixed amplitude‐normalizing scale factors based on the observed channel peak magnitudes. This latter normalization was not intended to estimate the thermal noise variance, but to balance the contributions of the pyruvate and lactate residuals so that the larger pyruvate signal did not dominate the joint objective function. The scale factors were computed once from the observed time courses and held fixed during the nonlinear optimization. Because this peak‐based scaling is a pragmatic channel‐balancing strategy rather than a formal noise‐likelihood model, all simulation analyses with known noise used the common RMS noise standard deviation directly.

Optimization was performed using bounded trust‐region reflective nonlinear least‐squares with multiple initializations. The solution with the lowest residual cost was retained. The kinetic parameters were constrained to the ranges and default initialization shown in Columns 2 and 3 of Table 1.

**TABLE 1:** Kinetic parameter bounds for NLLS fitting (second column) and synthetic training data generation (fourth column).

Parameter (units)	NLLS (min, max)	NLLS defaults	NN (min, max)
*k*_PL_ (s^−1^)	(10^−4^, 0.06)	0.05	(0.001, $k_{\mathrm{PL}}^{\max}$)
*k*_VE_ (s^−1^)	(10^−3^, 1)	0.15	(0.01, 0.3)
*v*_B_	(0.01, 0.5)	0.09	(0.005, 0.2)
*R*_1P_ (s^−1^)	(1/30, 1/30)	1/30	(1/60, 1/20)
*R*_1L_ (s^−1^)	(1/25, 1/25)	1/25	(1/55, 1/15)
*A*	($0.05{\;S}_{\mathrm{pyr},\max}^{\mathrm{obs}}$, $20{\;S}_{\mathrm{pyr},\max}^{\mathrm{obs}}$)	$S_{\mathrm{pyr},\max}^{\mathrm{obs}}$	(0.7, 1.3)
*t*_0_ (s)	*t*_start_ − 2TR ≤ *t*_0_ ≤ min(*t*_end_ − *t*_peak_)	max(*t*_start_ − TR, *t*_peak_ − 4)	(0.0, 2.0)
*α*	(1, 6)	3.0	(2.0, 4.0)
*β* (s^−1^)	(0.05, 2)	0.8	(0.6, 1.2)
SNR			(2, 100)

*Note:* The default initialization values for NLLS are shown in the third column. $t_{\text{peak}}$ is the time of the observed pyruvate maximum, $S_{\mathrm{pyr},\max}^{\mathrm{obs}}$. The NN training upper bound for $k_{\mathrm{PL}}$, $k_{\mathrm{PL}}^{\max}$, is set by default to 0.06 but extended to 0.2 for the TRAMP mouse to accommodate the expected higher $k_{\mathrm{PL}}$ values in the tumor.

Additional random initializations were sampled from physiologically plausible subranges within the specified bounds to reduce sensitivity to local minima. Flip angles were capped below 90°, and cosine values were numerically clipped to avoid singular RF‐depletion terms. The ODEs were integrated using an exponential update over each time interval, assuming constant input and apparent relaxation rate within that interval. The fitted output included ${\hat{k}}_{\mathrm{PL}}$, ${\hat{k}}_{\mathrm{VE}}$, ${\hat{v}}_{B}$, the fitted vascular input $P_{v}\left(t\right)$, and the fitted pyruvate and lactate signal curves for diagnostic inspection.

### 2.3 Synthetic Data Generator

An alternative to voxelwise nonlinear least‐squares fitting is to learn an amortized inverse map from dynamic hyperpolarized ^13^C pyruvate/lactate signals to kinetic parameters

(8) $$\left[S_{\mathrm{pyr}}\left(t\right),S_{\mathrm{lac}}\left(t\right)\right]\to \left[k_{\mathrm{PL}},k_{\mathrm{VE}},v_{B}\right]$$

To train this inverse model, we generated synthetic paired pyruvate and lactate time courses using the two‐compartment signal model described above. For each synthetic example, a latent vascular pyruvate input $P_{v}\left(t\right)$ was generated as a gamma‐variate‐like bolus curve over the protocol's acquisition time points using Equation (6) and values of $\left[A,\alpha ,\beta ,t_{0}\right]$ sampled from the ranges shown in Column 4 of Table 1. The resulting curve was peak‐normalized and scaled by the sampled peak amplitude. Using values of kinetic parameters, $\left[k_{\mathrm{PL}},k_{\mathrm{VE}},v_{B},R_{1,P},R_{1,L}\right]$, sampled from Column 4 of Table 1 and the generated $P_{v}\left(t\right)$, the extravascular pyruvate and lactate pools were evolved according to Equations (4) and (5) with metabolite‐specific apparent relaxation rates, $R_{1,\mathrm{pyr}}^{\mathrm{app}}\left(t\right)$ and $R_{1,\mathrm{lac}}^{\mathrm{app}}\left(t\right)$, computed according to Equation (3). The ODEs were integrated from zero initial conditions using a closed‐form exponential update over each time interval, assuming that the input and apparent relaxation rate were constant within each interval. This update was used to improve numerical stability relative to a simple Euler step. The measured pyruvate signal was generated after forward simulation using Equation (1) and the measured lactate signal with Equation (2).

Measurement noise was added after clean pyruvate and lactate curves were generated. For each synthetic example, a target SNR was drawn log‐uniformly from the configured SNR range (Table 1). The noise standard deviation was defined as the peak absolute pyruvate signal divided by the sampled target SNR, $\sigma =\max_{t}\left|S_{\mathrm{pyr}}\left(t\right)\right|/{\mathrm{SNR}}_{\text{target}}$. Independent zero‐mean Gaussian noise with standard deviation σ was then added to both pyruvate and lactate channels. Thus, the configured SNR corresponds approximately to the peak pyruvate SNR, while lactate SNR depends on the simulated lactate amplitude and the acquisition protocol. For each protocol, $10^{6}$ synthetic samples were generated. The generator returned the noisy paired pyruvate/lactate curves as an array of shape N×T×2, the corresponding labels, $\left[k_{\mathrm{PL}},k_{\mathrm{VE}},v_{B}\right]$, and the latent simulated pools $\left[P_{v}\left(t\right),P_{e}\left(t\right),L_{e}\left(t\right)\right]$ for diagnostic use.

Separate synthetic datasets were generated for each acquisition protocol evaluated in this study. They were a 16 time‐point variable‐flip angle (VFA) schedule ending at 90° with a TR of 2 s previously used in HP ^13^C study of a transgenic adenocarcinoma of mouse prostate (TRAMP) model [17], a 25 time‐point 15° constant‐flip‐angle (CFA) protocol with a TR of 2 s used for HP ^13^C of rat kidneys [17], and a 12 time‐point CFA protocol of 11° on pyruvate and 80° on lactate with a TR of 5 s used for HP ^13^C imaging in healthy human brains [4]. These acquisition protocols span a range of flip‐angle/TR designs (highly favorable, typical, and less favorable for lactate quantification), providing a broad testbed for $k_{\mathrm{PL}}$ estimation.

### 2.4 Neural Network Architecture

We implemented a compact hybrid multi‐head neural network (Figure 1C) to estimate voxelwise kinetic parameters from paired hyperpolarized ^13^C pyruvate and lactate time courses. This architecture was found to be more effective than temporal architectures such as 1D Convolutional Neural Networks or Recurrent Neural Networks which can overfit the extremely short HP ^13^C time courses [18]. The network was designed to learn the inverse mapping in Equation (8), using two complementary input representations: an amplitude‐preserving raw input branch and a pyruvate‐normalized input branch. This design was motivated by the different ways in which the kinetic parameters affect the observed signal (Figure 1B). The parameters $k_{\mathrm{PL}}$ and $k_{\mathrm{VE}}$ primarily influence the temporal shape of the lactate and pyruvate curves, including the lactate rise, peak, and decay and the pyruvate tail. In contrast, $v_{B}$ has a stronger dependence on signal amplitude because it controls the relative contribution of the vascular pyruvate component to the measured pyruvate signal. Therefore, the architecture was designed to use normalized temporal features for $k_{\mathrm{PL}}$ and $k_{\mathrm{VE}}$, while preserving amplitude information for $v_{B}$. For each voxel or synthetic sample, the input signal was represented as an array with shape T×2, where T is the number of acquired time points and the two channels correspond to pyruvate and lactate. Before input to the network, the paired time courses were converted into two flattened vectors of length 2T, with the raw branch defined as $x_{\mathrm{raw},i}=\kappa \left[S_{\mathrm{pyr},i}\left(t_{1}\right),\ldots ,S_{\mathrm{pyr},i}\left(t_{T}\right),S_{\mathrm{lac},i}\left(t_{1}\right),\ldots ,S_{\mathrm{lac},i}\left(t_{T}\right)\right]$, where κ is a global amplitude‐alignment factor. During synthetic training, κ=1. For in vivo or target‐domain inference, κ can be used to align the target signal amplitude distribution to the training distribution, $\kappa =P_{\text{train}}/P_{\text{target}}$, where $P_{\text{train}}$ and $P_{\text{target}}$ are robust percentiles, here the 99.9th percentile, of the voxelwise pyruvate peak amplitudes in the training and target datasets, respectively. This scaling is intended to reduce differences in absolute signal scale due to polarization, receiver gain, coil sensitivity, or reconstruction scaling. The normalized branch was generated from the scaled raw signal by dividing both metabolite channels by the voxelwise pyruvate peak, $p_{i}=\max_{t}S_{\mathrm{pyr},i}^{\mathrm{raw}}\left(t\right)$, to get $S_{\mathrm{pyr},i}^{\mathrm{norm}}\left(t\right)=S_{\mathrm{pyr},i}^{\mathrm{raw}}\left(t\right)/\max\left(p_{i},\epsilon \right)$ and $S_{\mathrm{lac},i}^{\mathrm{norm}}\left(t\right)=S_{\mathrm{lac},i}^{\mathrm{raw}}\left(t\right)/\max\left(p_{i},\epsilon \right)$.

Where $\epsilon =10^{-8}$ prevents division by zero. Thus, the normalized branch emphasizes temporal shape and relative lactate‐to‐pyruvate dynamics, while the raw branch preserves amplitude information. The NN was invariant to global multiplicative gain because target‐domain amplitude alignment canceled the gain in the amplitude‐preserving branch, while voxelwise peak normalization canceled it in the normalized branch.

The normalized input vector was passed through a shared fully connected feature extractor consisting of two hidden layers with 128 and 64 units, ReLU activations, and dropout with probability p=0.1 after each hidden layer. The resulting 64‐dimensional latent representation was used by two single‐output heads to estimate $k_{\mathrm{PL}}$ and $k_{\mathrm{VE}}$. Each of these heads consisted of a linear layer followed by a sigmoid activation. A third head estimated $v_{B}$ from the concatenation of the 64‐dimensional shared latent representation and the raw 2T‐dimensional input vector. This $v_{B}$ head consisted of a fully connected layer with 64 units, ReLU activation, dropout with probability p=0.1, a final linear layer, and a sigmoid activation.

The sigmoid outputs were mapped to physical parameter ranges using affine scaling. For a generic parameter $q\in \left\{k_{\mathrm{PL}},k_{\mathrm{VE}},v_{B}\right\}$, the network output was $q=q_{\min}+\sigma \left(z_{q}\right)\left(q_{\max}-q_{\min}\right)$, where $z_{q}$ is the corresponding pre‐sigmoid network output, σ(·) is the sigmoid function, and $q_{\min}$ and $q_{\max}$ are taken from the ranges defined in Table 1. This constrained‐output formulation ensured that all network predictions remained within the physiologic ranges used during simulation. The implementation stores these lower bounds and spans as model buffers so that they remain associated with the model during CPU/GPU transfer. The model was trained using an unweighted mean‐squared‐error loss over the three output parameters,

(9) $$L=\frac{1}{N}\sum_{i=1}^{N}\left[{\left({\hat{k}}_{\mathrm{PL},i}-k_{\mathrm{PL},i}\right)}^{2}+{\left({\hat{k}}_{\mathrm{VE},i}-k_{\mathrm{VE},i}\right)}^{2}+{\left({\hat{v}}_{B,i}-v_{B,i}\right)}^{2}\right]$$

and optimized using adaptive moment (Adam) estimation [19] with learning rate $10^{-3}$. For each protocol, the synthetic dataset was randomly split into training, validation, and test sets using a 70/15/15 split with random seed 42, and the same indices were applied to the raw branch, normalized branch, and parameter labels. Training used early stopping based on validation loss with patience of 50 epochs and a minimum improvement threshold of $10^{-6}$. The model state with the lowest validation loss was restored before final testing.

### 2.5 Model Evaluation

After training, each protocol‐specific neural network was evaluated on simulated pyruvate/lactate time courses generated with the corresponding acquisition timing, flip‐angle schedule, relaxation assumptions, and kinetic‐parameter ranges. For neural‐network inference, paired pyruvate and lactate curves were preprocessed using the same hybrid input procedure used during training: an amplitude‐preserving raw branch and a pyruvate‐peak‐normalized branch. For each target dataset, the raw branch was globally amplitude‐aligned to the training distribution using the robust pyruvate‐peak scaling factor described above, and both branches were then flattened and passed through the trained network to estimate $k_{\mathrm{PL}}$, $k_{\mathrm{VE}}$, and $v_{B}$. For the primary comparison, NLLS was implemented using the same gamma‐variate vascular‐input model used to generate synthetic training data. Thus, $k_{\mathrm{PL}}$, $k_{\mathrm{VE}}$, $v_{B}$, and the vascular‐input parameters were estimated jointly from the pyruvate and lactate time courses. This same NLLS formulation was used for simulated and in vivo comparisons. For simulated evaluations, clean pyruvate/lactate curves were first generated and then corrupted with independent zero‐mean Gaussian noise. The same root‐mean‐square noise amplitude was applied to both metabolites within a sample or condition, so differences between reported pyruvate and lactate SNR reflected differences in clean signal amplitudes rather than different noise amplitudes. Pyruvate and lactate SNR were therefore reported as ${\mathrm{SNR}}_{\mathrm{pyr}}=\max_{t}|S_{\mathrm{pyr},\text{clean}}\left(t\right)|/\sigma$ and ${\mathrm{SNR}}_{\mathrm{lac}}=\max_{t}|S_{\mathrm{lac},\text{clean}}\left(t\right)|/\sigma$, where σ is the Gaussian noise standard deviation. This equal‐noise convention was used for the primary simulated agreement and perturbation analyses. Model‐truth agreement was quantified using complementary metrics. Explained variance was summarized using the coefficient of determination,

(10) $$R^{2}=1-\frac{\sum_{i}{\left({\hat{y}}_{i}-y_{i}\right)}^{2}}{\sum_{i}{\left(y_{i}-\overset{`}{y}\right)}^{2}}$$

where $y_{i}$ is the simulated reference value and ${\hat{y}}_{i}$ is the estimated value. Negative $R^{2}$ values were allowed and indicate performance worse than predicting the sample mean. Error magnitude was summarized using root‐mean‐square error.

(11) $$\text{RMSE}=\sqrt{\frac{1}{N}\sum_{i}{\left({\hat{y}}_{i}-y_{i}\right)}^{2}}$$

Bias was assessed using Deming regression of predicted versus true parameter values, which accounts for uncertainty in both axes. The Deming slope and intercept were used to assess proportional and constant bias, respectively, with bootstrap confidence intervals for the slope. Bootstrap resampling was also used to estimate uncertainty in $R^{2}$.

Sensitivity to acquisition and physics perturbations was evaluated using three simulation experiments. First, SNR sensitivity was tested by generating clean curves and adding equal‐rms Gaussian noise over a range of target pyruvate SNR values. Second, relaxation sensitivity was tested by varying pyruvate/lactate $T_{1}$ pairs from 20/15 to 60/55 s. Third, flip‐angle sensitivity was tested by perturbing both pyruvate and lactate flip‐angle schedules by offsets from −4° to +4°. The perturbation scripts evaluated NN and NLLS using the nominal training assumptions during fitting/inference, thereby testing robustness to mismatch between the true acquisition/relaxation conditions and the assumed model. For each perturbation condition, loss of accuracy was summarized using $R^{2}$ and its bootstrap standard deviations for each parameter.

### 2.6 In Vivo Inference

For in vivo datasets, reconstructed pyruvate and lactate dynamic images were loaded as paired NIfTI time series with time as the final array dimension. For NN inference, the pyruvate and lactate time courses were reshaped into voxelwise T×2 inputs, robustly amplitude‐aligned to the training distribution using the 99.9th percentile of voxelwise pyruvate peak amplitudes and passed through the trained network. The resulting $k_{\mathrm{PL}}$, $k_{\mathrm{VE}}$, and $v_{B}$ predictions were reshaped back into image volumes and saved as NIfTI parameter maps. For comparison, voxelwise NLLS maps were generated using the gamma‐variate AIF NLLS routine with the same protocol‐specific timing and flip‐angle schedules. The AUC for each metabolite was computed from the definition,

(12) $${\mathrm{AUC}}_{m}=\int_{0}^{T_{\mathrm{end}}}{\left[S_{m}\left(t\right)\right]}_{+}\;\mathrm{dt},m\in \mathrm{Lac},\mathrm{Pyr}$$

where $S_{m}\left(t\right)$ is the reconstructed signal time course for metabolite m, $T_{\mathrm{end}}$ is the final acquired dynamic time point, and ${\left[\cdot \right]}_{+}$ denotes nonnegativity enforcement before integration, implemented by trapezoidal integration over the acquired time points, and the AUC ratio was computed as

(13) $${\mathrm{AUC}}_{\mathrm{Lac}/\mathrm{Pyr}}=\frac{{\mathrm{AUC}}_{\mathrm{Lac}}}{\max\left({\mathrm{AUC}}_{\mathrm{Pyr}},\epsilon \right)}$$

Summary statistics for AUC and parameter maps, including foreground‐voxel summary statistics, were saved for quality control, and a montage of the central slices of the images was generated for visual comparison.

### 2.7 Investigating Whether $k_{\mathrm{PL}}$ Provides Information Beyond AUC Ratio

To test whether estimated $k_{\mathrm{PL}}$ contains information beyond the static Lac/Pyr AUC ratio, we performed a matched‐ratio simulation experiment. Paired negative and positive samples were generated with different true pyruvate‐to‐lactate conversion rates but matched Lac/Pyr AUC ratios. The negative class used $k_{\mathrm{PL}}^{\text{true}}=0.120\;s^{-1}$, and the positive class used $k_{\mathrm{PL}}^{\text{true}}=0.144\;s^{-1}$. For each negative sample, a corresponding positive sample was generated by resampling physiologic and acquisition‐related confounds until the positive sample's AUC ratio matched the negative sample within a specified tolerance, ∣ΔAUCratio∣≤0.01. The resampled confounds included $v_{B}$, $k_{\mathrm{VE}}$, bolus/acquisition‐window timing shift, AIF shape parameters, and independent noise realizations. For each retained matched pair, Lac/Pyr AUC ratio and estimated $k_{\mathrm{PL}}$ were used as classifiers for the positive versus negative class. Discriminability was quantified using the Area Under the Receiver Operating Characteristic (AUROC) for both metrics. Preservation of metabolic sensitivity under AUC‐ratio matching was additionally summarized using paired differences, $\Delta {\mathrm{AUC}}_{\text{ratio}}={\mathrm{AUC}}_{\text{ratio}}^{\text{positive}}-{\mathrm{AUC}}_{\text{ratio}}^{\text{negative}}$ and $\Delta {\hat{k}}_{\mathrm{PL}}={\hat{k}}_{\mathrm{PL}}^{\text{positive}}-{\hat{k}}_{\mathrm{PL}}^{\text{negative}}$. If the matching procedure is successful, $\Delta {\mathrm{AUC}}_{\text{ratio}}$ should be centered near zero. A positive shift in $\Delta {\hat{k}}_{\mathrm{PL}}$ despite matched AUC ratios indicates that the estimator preserves kinetic information not captured by the integrated Lac/Pyr ratio alone. The experiment generated ROC curves and paired‐difference histograms for this comparison.

## 3 Results

The neural network's training took about 28 min on a Windows 10 desktop (Xeon E5‐2699A CPU). Processing the clinical dataset, which had a volume of 24 × 24 × 24 voxels, with the trained NN was essentially instantaneous (70 ms) but took 2 h 56 min using NLLS fitting on the same CPU. We first evaluated synthetic‐data accuracy under the VFA protocol. Figure 2 compares NN and NLLS estimation on synthetic HP ^13^C data generated over the SNR range in Table 1 using the VFA scheme. NLLS shows good agreement for $k_{\mathrm{PL}}$ ($R^{2}=0.80\pm 0.08$) but poorer agreement for $k_{\mathrm{VE}}$ ($R^{2}=0.22\pm 0.15$) and $v_{B}$ ($R^{2}=-0.19\pm 0.23$). By contrast, the NN achieves higher coefficients of determination: $R^{2}=0.93\pm 0.01$ ($k_{\mathrm{PL}}$), 0.83±0.02 ($k_{\mathrm{VE}}$), and 0.66±0.06 ($v_{B}$). Figure 3 illustrates representative predicted trajectories to simulated data, generated with the VFA protocol, using NN (solid blue) and NLLS (dashed red). Note that NLLS is explicitly optimized to minimize the curve residual, whereas the NN is optimized to estimate kinetic parameters robustly from noisy curves. Therefore, residual goodness‐of‐fit is expected to favor NLLS and is not the primary endpoint. Both methods also fit the lactate data well despite the poorer prediction of $k_{\mathrm{VE}}$ and $v_{B}$ by NLLS because although those parameters are weakly identifiable, they are also mutually compensatory and similar curve fits can coexist with substantially different parameter accuracy.

**FIGURE 2:**
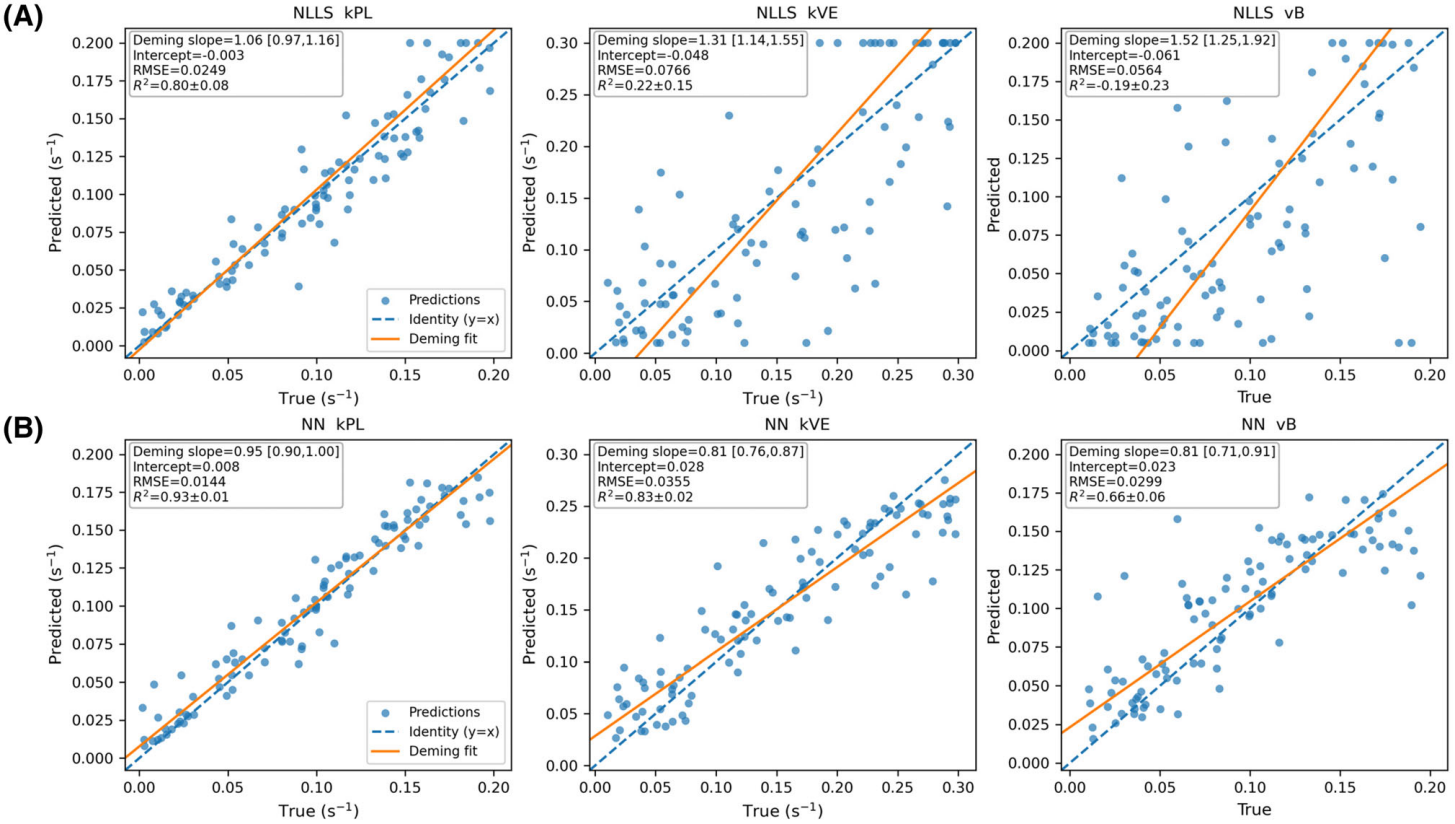
Agreement between **true** and **predicted** kinetic parameters for a two‐compartment HP‐^13^C model using (A) non‐linear least squares (NLLS) and (B) a hybrid multi‐head neural network. Each point is one simulated voxel (*n* = 100 randomly selected time‐series pairs). Blue dashed line: Identity y=x. Reported metrics are $R^{2}$ (with bootstrap SD), regression slope/intercept, and RMSE. Parameter units: $k_{\mathrm{PL}},k_{\mathrm{VE}}$ in s^−1^; $v_{B}$ unitless fraction. Simulations used a 16‐step variable‐flip angle schedule. Note that the NLLS is explicitly optimized to minimize the curve residual, whereas the NN is optimized to estimate kinetic parameters robustly from noisy curves. Therefore, residual goodness‐of‐fit is expected to favor NLLS and is not the primary endpoint. Axes are identically windowed across methods for each parameter.

**FIGURE 3:**
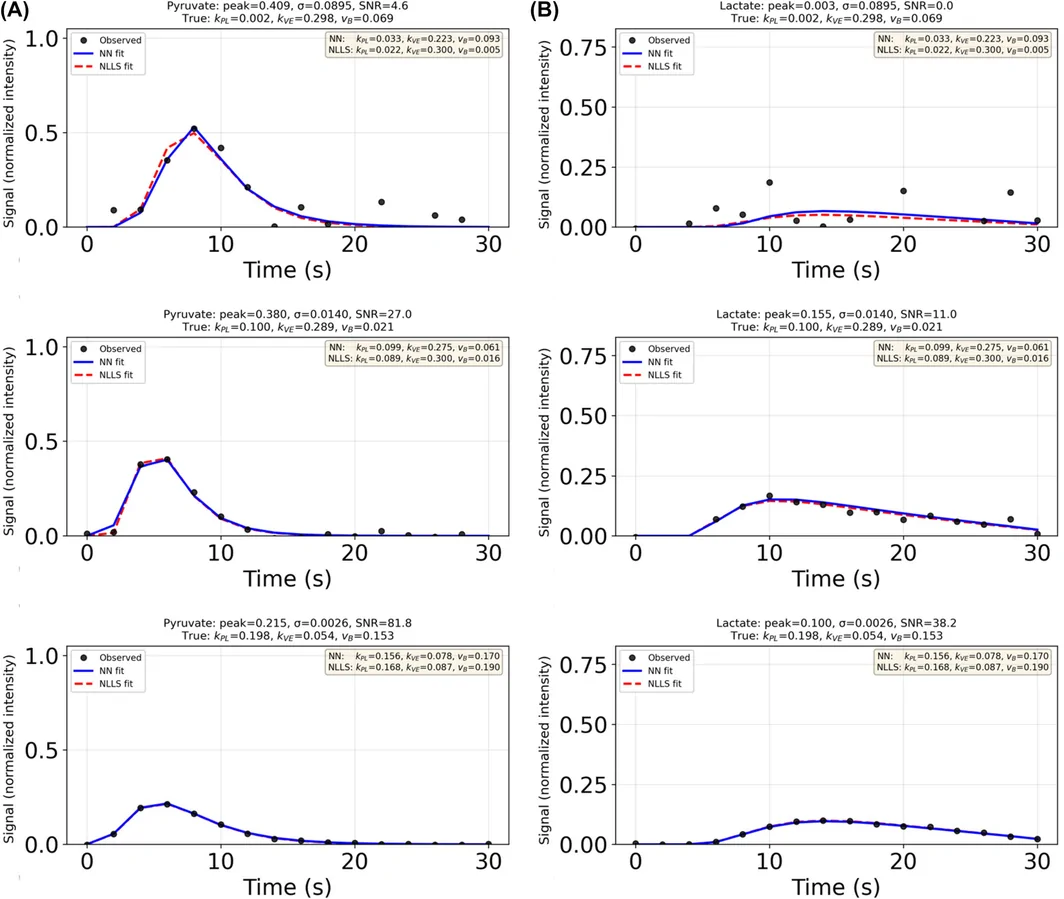
Selected voxel‐level simulations using estimated kinetic parameters for (A) **pyruvate** and (B) **lactate** time courses generated with a 16‐timepoint VFA schedule (TR = 2 s; $T_{1P}/T_{1L}=30/25$ s); Black dots: Observed data; **solid blue**: NN forward simulation using the gamma AIF model; **red dashed**: NLLS fit. Panels report true parameters and method estimates $\left(k_{\mathrm{PL}},k_{\mathrm{VE}}\;\left[s^{-1}\right],v_{B}\right)$ and per‐panel RMSE. Identical axis ranges are used within each metabolite to enable visual comparison.

$R^{2}$ values for $k_{\mathrm{PL}}$ were slightly lower under the CFA scheme that used flip angles of 15° pyruvate/lactate, reflecting the reduced signal preservation by this scheme compared to VFA. However, the $R^{2}$ values were higher for NN across all three metabolic parameters (Figures S2A and S2B).

$R^{2}$ values for $k_{\mathrm{PL}}$ estimation were lowest for the 11°/80° CFA scheme (NN $k_{\mathrm{PL}}$ $R^{2}$ = 0.68±0.05, NLLS $k_{\mathrm{PL}}$ $R^{2}$ = 0.02±0.25) (Figures S3A and S3B). This protocol was designed to enhance lactate visualization, and the high lactate flip angle rapidly depletes lactate magnetization, reducing the temporal information available for $k_{\mathrm{PL}}$ estimation. Consequently, this scheme is better suited to lactate‐weighted contrast than to quantitative $k_{\mathrm{PL}}$ mapping. It can be observed that the $R^{2}$ values for $k_{\mathrm{VE}}$ ($R^{2}$ = 0.84±0.05) and $v_{B}$ ($R^{2}$ = 0.69±0.07) estimation by NN remained high for this protocol, since these depend on delivery/exchange of pyruvate.

We then quantified robustness to sequence/physics perturbations and tested whether the NN captures information beyond the static AUC Lac/Pyr ratio. Figure 4 summarizes robustness to $T_{1}$, SNR, and flip‐angle error. Across all conditions, the NN maintains comparable or higher $R^{2}$ for $k_{\mathrm{PL}}$, $k_{\mathrm{VE}}$, and $v_{B}$. Consistent with the results of Sun et al. [20], performance worsens rapidly when pyruvate SNR fell below approximately 20, however NN performance was improved relative to NLLS (Figure 4A).

**FIGURE 4:**
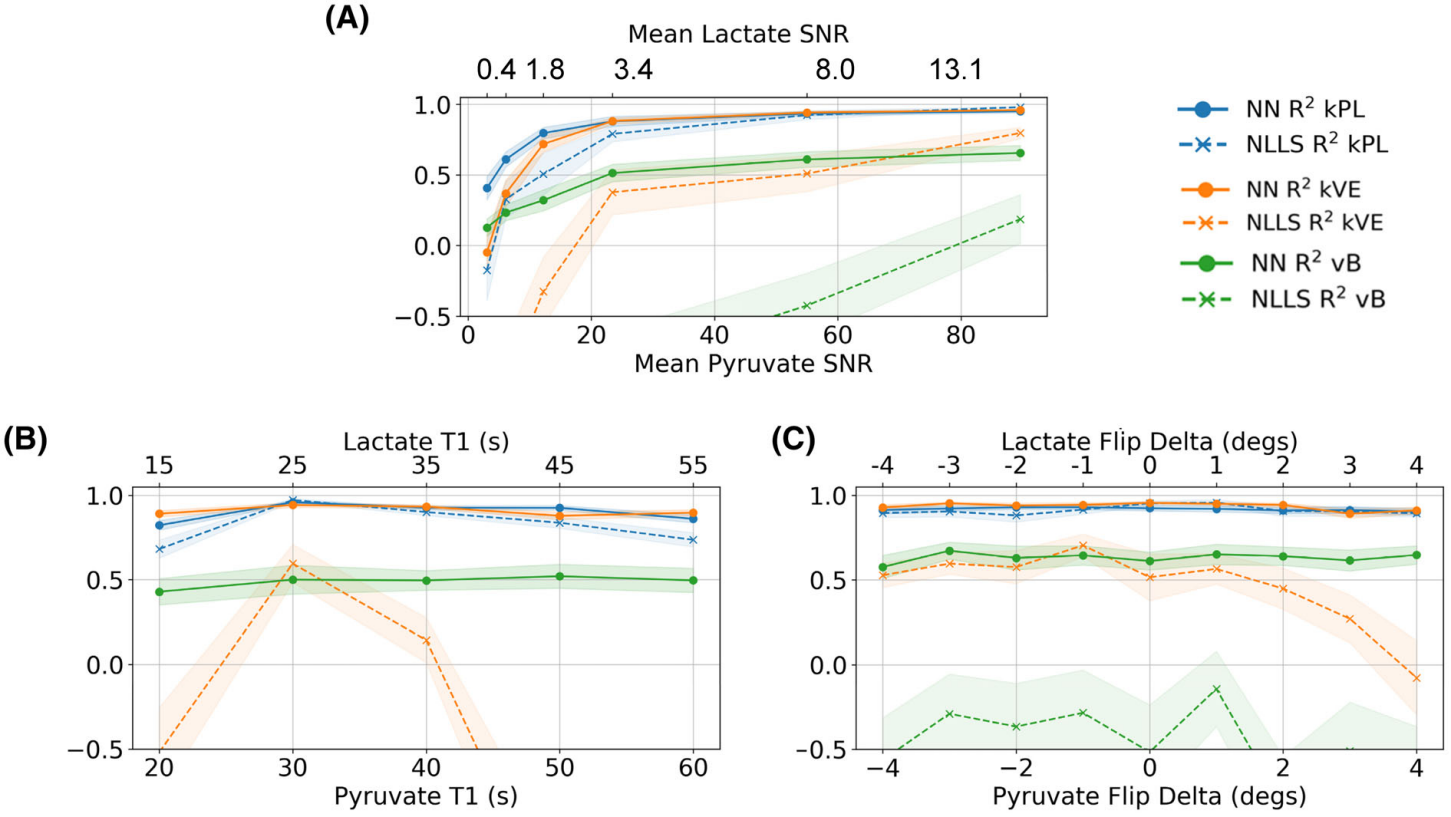
Sensitivity of parameter recovery to sequence/physics factors in a two‐compartment HP‐^13^C model. Mean $R^{2}$ (lines) ± SD (shaded, bootstrap B=200, n=100 per setting) for **NN** (solid) and **NLLS** (dashed) when varying (A) Pyruvate/Lactate SNR, (B) Pyruvate/Lactate $T_{1}$ (s), and (C) flip‐angle error (deg, relative to nominal). Parameters: $k_{\mathrm{PL}},k_{\mathrm{VE}}\;\left[s^{-1}\right],v_{B}$ (unitless). Negative $R^{2}$ at low SNR/large errors indicate NLLS instability; NN degrades more gracefully (P and L were varied in lockstep at each setting). The range of values tested were $T1_{P/L}=\left\{20/15\;\text{to}\;60/55\right\}$ s; SNR = 2–100; ΔFA = ±4°.

Figure 5A shows ROC curves for classifying two kinetic conditions ($k_{\mathrm{PL}}^{\text{true}}$ = 0.120 vs. 0.144 s^−1^) using either the conventional AUC‐based Lac/Pyr surrogate (${\mathrm{AUC}}_{\mathrm{Lac}}/{\mathrm{AUC}}_{\mathrm{Pyr}}$) or $k_{\mathrm{PL}}$ (${\hat{k}}_{\mathrm{PL}}$). Because the positive class was constructed to match ${\mathrm{AUC}}_{\mathrm{Lac}}/{\mathrm{AUC}}_{\mathrm{Pyr}}$ to the negative class across resampled delivery/timing/AIF confounds, the Lac/Pyr surrogate becomes non‐discriminative (AUROC ≈ 0.50). In contrast, ${\hat{k}}_{\mathrm{PL}}$ remains sensitive to the imposed metabolic difference (AUROC ≈ 0.86 for NLLS and AUROC ≈ 0.76 for NN). This conclusion is further supported by paired‐difference histograms for each matched pair (Δ = positive − negative) showing that the AUC ratio differences are centered near zero (median |Δ| < 0.005), while ${\hat{k}}_{\mathrm{PL}}$ differences remain shifted positive, indicating sensitivity to metabolic conversion even when ${\mathrm{AUC}}_{\mathrm{Lac}}/{\mathrm{AUC}}_{\mathrm{Pyr}}$ is held constant (Figure 5B,C).

**FIGURE 5:**
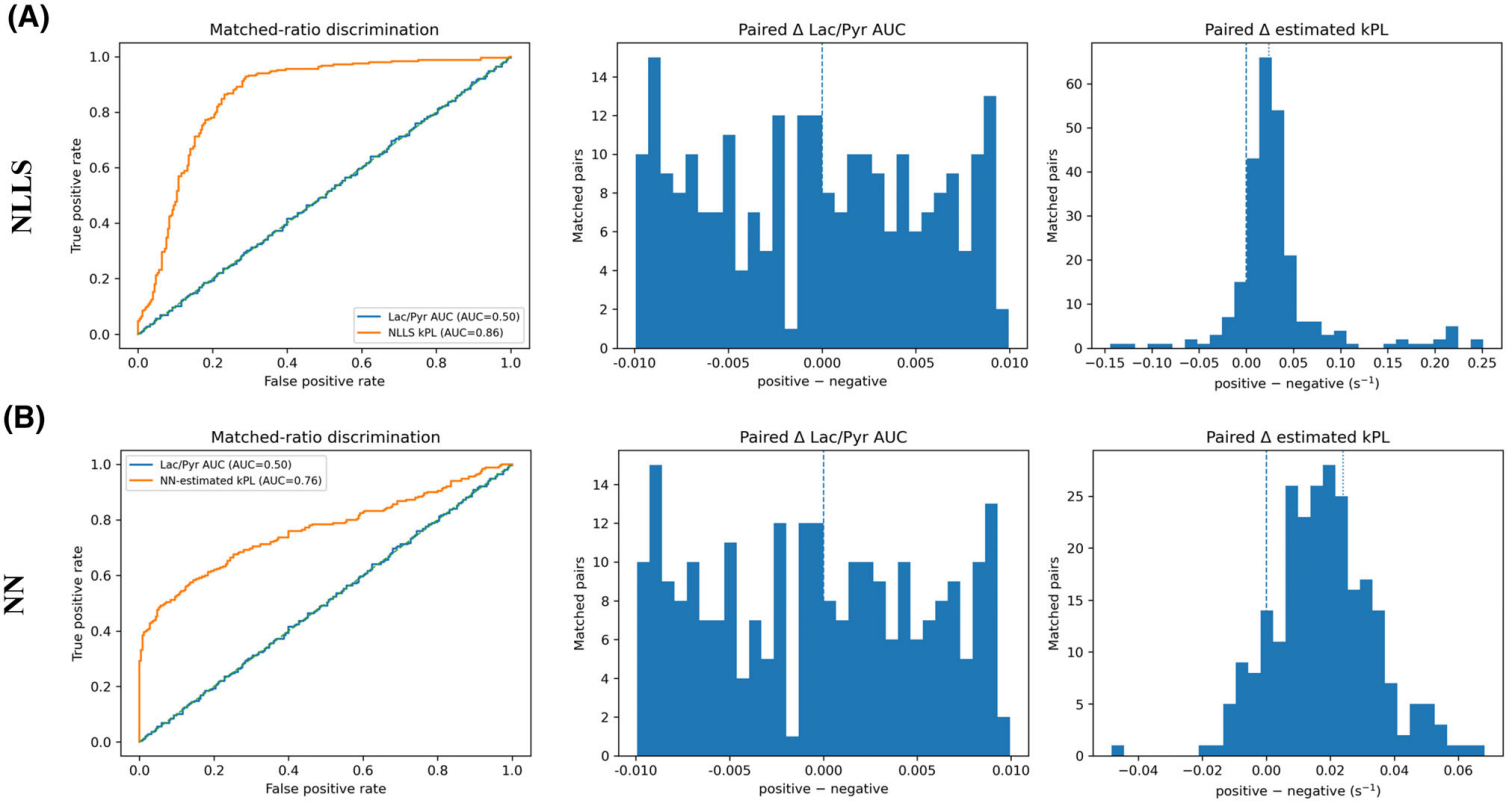
Matched‐ratio discrimination experiment demonstrates information beyond Lac/Pyr. (A) ROC curves for classifying two kinetic conditions ($k_{\mathrm{PL}}^{\text{true}}$ = 0.120 vs. 0.144 s^−1^) using either the conventional AUC‐based Lac/Pyr surrogate (${\mathrm{AUC}}_{\text{Lactate}}/{\mathrm{AUC}}_{\text{Pyruvate}}$) or model‐estimated $k_{\mathrm{PL}}$. Positive samples were explicitly resampled over delivery/timing/AIF confounds until their ${\mathrm{AUC}}_{\text{Lactate}}/{\mathrm{AUC}}_{\text{Pyruvate}}$ matched the corresponding negative sample within tolerance (|Δ(${\mathrm{AUC}}_{\text{ratio}}$)| ≤ 0.01), forcing the ratio‐based metric to be non‐discriminative by design. In this matched setting, ${\mathrm{AUC}}_{\text{Lactate}}/{\mathrm{AUC}}_{\text{Pyruvate}}$ yields chance performance (AUROC ≈ 0.50), whereas ${\hat{k}}_{\mathrm{PL}}$ remains discriminative (AUROC ≈ 0.86 for NLLS and AUROC ≈ 0.76 for NN). (B) Paired‐difference histograms for each matched pair (Δ = positive − negative) showing that the AUC ratio differences are centered near zero (median |Δ| ≈ 0.005), while ${\hat{k}}_{\mathrm{PL}}$ differences remain shifted positive, indicating sensitivity to metabolic conversion even when Lac/Pyr is held constant.

Finally, we examined in vivo parameter maps in mouse, rat kidney, and human brain datasets. Figure 6 shows metabolic parameter mapping in a TRAMP mouse tumor. The T1‐weighted image delineates tumor anatomy while pyruvate and lactate AUC maps highlight delivery/exchange and metabolic activity respectively. Despite good performance on parameter recovery in simulations (Figure 2), the NLLS maps show boundary accumulation and spatial discontinuity suggesting a model mismatch in vivo. On the other hand, the NN‐derived maps show spatial coherence, with the $k_{\mathrm{PL}}$ map showing spatial correspondence to the Lac/Pyr AUC ratio map, and the $v_{B}$ map showing spatial correspondence to the pyruvate AUC map consistent with its expected sensitivity to vascular delivery. This visual observation is quantified in Table S3, which shows the Spearman correlation coefficients (ρ) between the parameter and the AUC maps and the percentage of voxels within 1% of the lower or upper voxel values. NN shows higher ρ and small percentage of boundary voxels, supporting the visual observation that the NN produced more stable in vivo parameter maps than voxelwise NLLS fitting.

**FIGURE 6:**
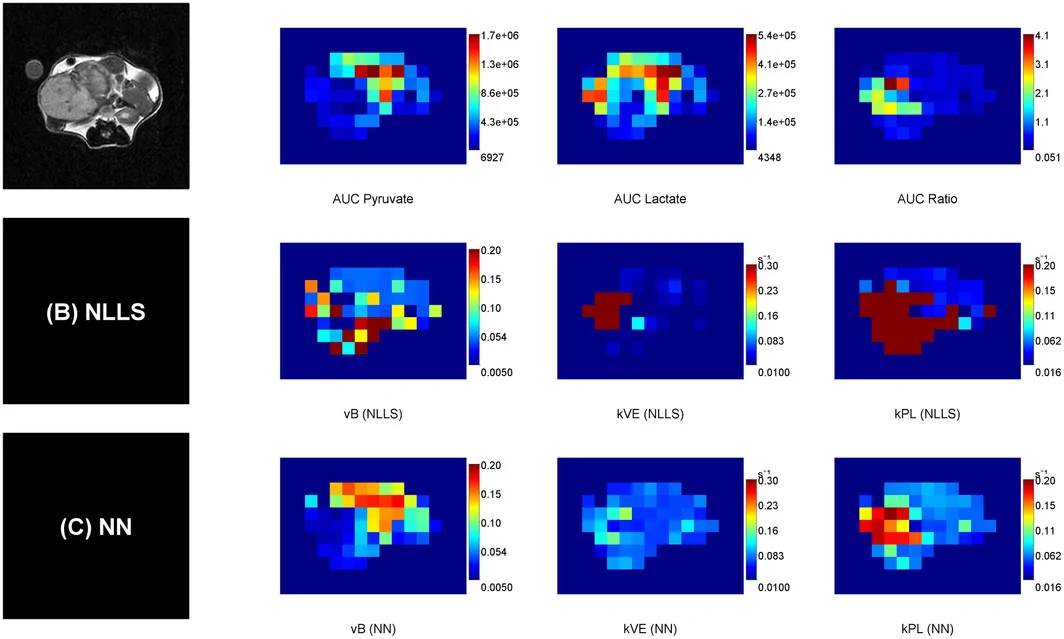
TRAMP mouse tumor HP‐^13^C MRI: Anatomy, AUCs, and kinetic maps. Top: T1‐weighted reference, AUC_pyr_ and AUC_lac_ (a.u.), and unitless AUC_lac_/AUC_pyr_ ratio from dynamic HP‐^13^C MRI (same slice/registration). Middle: Voxelwise parameters from **NLLS**. Bottom: Corresponding NN maps. Colorbars share identical windows across methods ($v_{B}$: 0–0.20; $k_{\mathrm{VE}}$: 0–0.35 s^−1^; $k_{\mathrm{PL}}$: 0–0.2 s^−1^).

Figure 7 compares the results of parameter mapping for the kidneys in a normal rat using NLLS and NN. The T1‐weighted image is provided for anatomic localization; pyruvate and lactate AUC maps are shown. Using identical display windows for both methods, the $k_{\mathrm{PL}}$ maps derived using NLLS and NN show spatial correspondence with the Lac/Pyr AUC ratio map. However, there is a large discrepancy between the NN and NLLS depictions of the $v_{B}$ and $k_{\mathrm{VE}}$ maps with NLLS showing boundary‐hitting, which would be consistent with the low $R^{2}$ values observed in the simulation results. The NN depictions of the same maps show spatial correspondence with the pyruvate AUC map which would be consistent with their sensitivity to delivery and exchange. The Spearman correlation coefficients and boundary voxel percentage in Table S4 confirm the visual observation.

**FIGURE 7:**
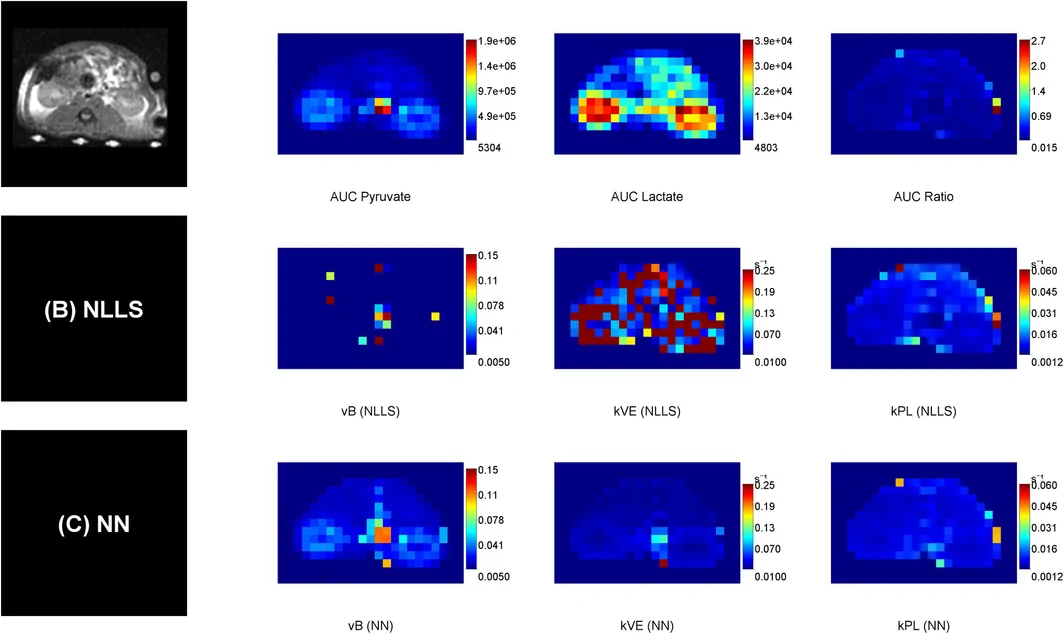
Rat kidneys HP‐^13^C MRI: Anatomy, AUCs, and kinetic maps. Top: T1‐weighted reference, AUC_pyr_ and AUC_lac_ (a.u.), and unitless AUC_lac_/AUC_pyr_ ratio from dynamic HP‐^13^C MRI (same slice/registration). Middle: Voxelwise parameters from **NLLS**. Bottom: Corresponding NN maps. Colorbars share identical windows across methods ($v_{B}$: 0–0.15; $k_{\mathrm{VE}}$: 0–0.25 s^−1^; $k_{\mathrm{PL}}$: 0–0.06 s^−1^).

Figure 8 presents images of a healthy human brain acquired using the (11°/pyruvate, 80°/lactate) protocol. All NLLS‐derived maps appear to show boundary saturation and spatial discontinuity; however, the NN maps show good spatial correlation to the AUC and anatomical maps. Additional clinical exams are shown in Figures S4A and S4B. Increasing the $k_{\mathrm{PL}}$ upper bound during NLLS fitting reduced boundary saturation but did not recover the spatial pattern observed with the NN. Instead, the NLLS maps changed substantially with the imposed bound and developed isolated extreme estimates, indicating that the NLLS map is not merely a rescaled NN map (Table S5 and Figure S5).

**FIGURE 8:**
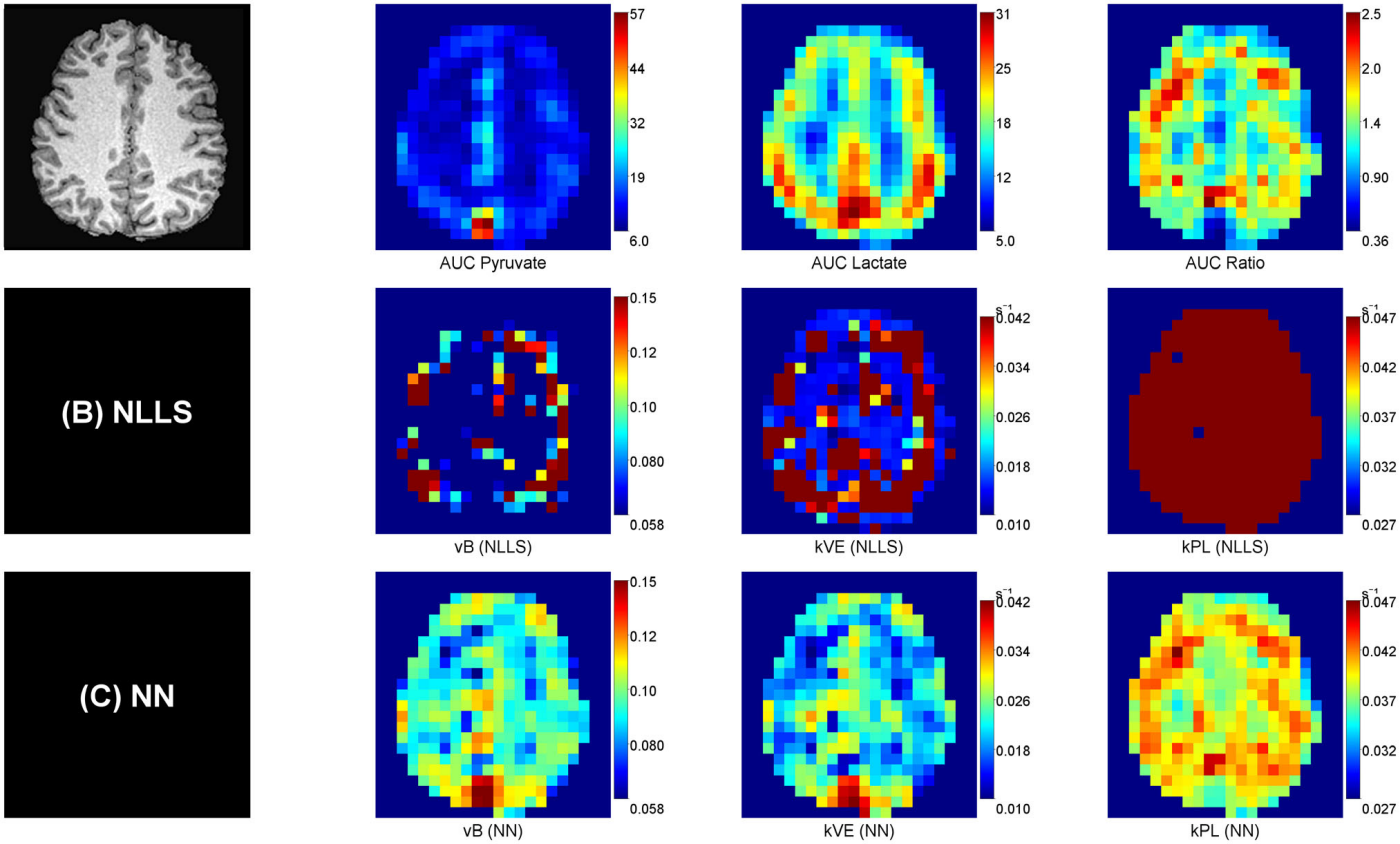
Brain HP‐^13^C MRI: Anatomy, AUCs, and kinetic maps. Top: T1‐weighted reference (same slice/registration), AUC_pyr_ and AUC_lac_ (a.u.), and unitless AUC_lac_/AUC_pyr_ ratio. Middle: Voxelwise parameters from NLLS. Bottom: Corresponding NN maps. Colorbars use identical windows across methods to enable fair comparison ($v_{B}$: 0–0.15; $k_{\mathrm{VE}}$: 0–0.04 s^−1^; $k_{\mathrm{PL}}$: 0–0.05 s^−1^).

## 4 Discussion and Conclusion

This study demonstrates that a compact, physics‐informed neural network (NN) trained on simulated hyperpolarized ^13^C pyruvate/lactate dynamics can provide more robust voxelwise kinetic parameter estimates than conventional nonlinear least‐squares (NLLS) fitting, particularly under low‐SNR and weakly identifiable conditions. The central challenge is that the inverse problem is highly coupled: the same pyruvate/lactate time courses can often be reproduced by compensatory changes in delivery, exchange, vascular fraction, and input‐function parameters. In our voxelwise gamma‐variate formulation, this flexibility accommodates regional heterogeneity in bolus arrival, perfusion, and washout, but it also increases the degrees of freedom available to NLLS. Consistent with this interpretation, the supplementary identifiability analysis showed that the seven‐parameter NLLS formulation was locally full rank but practically ill‐conditioned, with substantial covariance coupling among $k_{\mathrm{VE}}$, $v_{B}$, and the AIF amplitude/shape parameters. Thus, the poor recovery of $k_{\mathrm{VE}}$ and $v_{B}$ by NLLS does not necessarily indicate a failure of the forward model; rather, it reflects the limited information content of short, noisy HP ^13^C time courses when multiple delivery‐related parameters are estimated simultaneously. The NN mitigates this instability by acting as a regularized inverse estimator constrained by a physiologic synthetic training distribution.

A consistent finding was that parameter recovery depended strongly on the excitation scheme. The variable‐flip‐angle (VFA) acquisition produced the best simulation performance, consistent with preservation of informative late‐time pyruvate and lactate dynamics. In contrast, the 11° pyruvate/80° lactate scheme reduced $k_{\mathrm{PL}}$ accuracy, most likely because the high lactate flip angle rapidly depletes lactate magnetization and limits the temporal information available for estimating conversion. The relatively stronger NN recovery of $k_{\mathrm{VE}}$ and $v_{B}$ under this protocol likely reflects preservation of the pyruvate driver by the low pyruvate flip angle, whereas $k_{\mathrm{PL}}$ remains more dependent on the depleted lactate time course. NLLS did not show the same robustness, consistent with its greater sensitivity to parameter compensation and poorly conditioned residual surfaces. These results support the use of VFA schemes for kinetic mapping when feasible, while also emphasizing that no estimator can recover information that is not adequately encoded by the acquisition. Recent Fisher‐information‐based optimization studies similarly indicate that appropriately designed VFA schedules can reduce kinetic‐parameter uncertainty and that $B_{1}$ variation should be incorporated to avoid biased parameter estimates [21].

The in vivo examples further support the practical advantage of the NN. Both NLLS and NN produced visually coherent $k_{\mathrm{PL}}$ maps in some settings, particularly for the 15° CFA protocol, but the NN more consistently generated physiologically plausible maps across all parameters, protocols, and datasets. NN‐derived delivery‐sensitive parameters preserved the expected spatial correspondence with pyruvate AUC, while $k_{\mathrm{PL}}$ maps remained spatially related to the conventional ${\mathrm{AUC}}_{\mathrm{Lac}/\mathrm{Pyr}}$ contrast. These spatial relationships provide useful internal consistency checks, but they should not be interpreted as independent validation because AUC metrics are themselves affected by delivery, relaxation, flip‐angle depletion, receive sensitivity, and bolus timing.

The relationship between $k_{\mathrm{PL}}$ and ${\mathrm{AUC}}_{\mathrm{Lac}/\mathrm{Pyr}}$ therefore requires careful interpretation. Consistent with prior studies, $k_{\mathrm{PL}}$ maps were spatially related to AUC‐derived metabolic contrast. However, the matched‐ratio experiment showed that when ${\mathrm{AUC}}_{\mathrm{Lac}/\mathrm{Pyr}}$ was intentionally made non‐discriminative across conditions with different $k_{\mathrm{PL}}$, model‐based $k_{\mathrm{PL}}$ estimates retained discriminatory information. This supports kinetic modeling when the goal is to separate metabolic conversion from delivery, timing, and acquisition‐dependent signal weighting. At the same time, ${\mathrm{AUC}}_{\mathrm{Lac}/\mathrm{Pyr}}$ remains valuable as a simple model‐free quality‐control metric and spatial comparator.

The matched‐ratio experiment also clarifies the scope of the NN advantage. In the ideal model‐matched setting, NLLS‐derived $k_{\mathrm{PL}}$ showed higher discrimination than NN‐derived $k_{\mathrm{PL}}$ (AUROC ≈ 0.86 for NLLS and AUROC ≈ 0.76 for NN). This is expected because the data were generated from the same forward model fitted by NLLS, with fixed relaxation and flip‐angle assumptions. Under such ideal conditions, NLLS should approach the maximum‐likelihood solution for Gaussian noise. The NN advantage is therefore not universal; rather, it reflects a trade‐off. The NN sacrifices some ideal‐case sharpness in exchange for substantially improved stability, speed, and spatial coherence in low‐SNR or practically ill‐conditioned regimes.

Several limitations should be emphasized. First, temporally resolved HP ^13^C acquisitions are sensitive to bolus timing. If pyruvate arrival is delayed, blunted, or poorly sampled, the measured dynamics may contain insufficient information for reliable kinetic estimation, regardless of whether NLLS or NN inference is used. This limitation also applies to model‐free AUC analysis: although AUC ratios may be less sensitive to modest timing variation than full kinetic fitting, they still require adequate temporal coverage of pyruvate delivery and lactate production. Kinetic modeling should therefore be accompanied by acquisition‐level quality control, including assessment of bolus arrival, peak timing, and coverage of the relevant metabolite curves.

Second, synthetic data training introduces the risk of distribution shift. The NN behaves as a high‐dimensional interpolator within the physiologic and acquisition space represented during training; if target data fall outside that support, the resulting maps may appear smooth and plausible while still being biased. We observed this during model development when a mismatched $k_{\mathrm{PL}}$ training range produced systematic bias in vivo. This motivated the use of protocol‐specific physiologic training ranges that better represented the expected target values. Future work should refine these priors using independent biological measurements, repeatability data, and larger multi‐cohort datasets.

Finally, the current in vivo evaluation provides physiologic plausibility rather than absolute biochemical validation. The observed agreement between NN‐derived maps and AUC‐based spatial patterns supports internal consistency, but quantitative validation against independent standards such as biopsy‐derived metabolic activity, orthogonal metabolic imaging, or same‐day repeatability remains necessary before the method can be recommended for quantitative clinical use.

In conclusion, deep learning‐based HP ^13^C MRI kinetic modeling provides rapid, regularized voxelwise parameter estimation and improves robustness relative to NLLS in low‐SNR and weakly identifiable regimes. The results show that NN‐based inference can preserve kinetic information beyond static Lac/Pyr AUC ratios while producing spatially coherent parameter maps across preclinical and clinical datasets. Its reliability, however, depends on the fidelity and coverage of the synthetic training distribution. When the simulated training space matches the acquisition physics and plausible physiology of the target application, physics‐informed deep learning offers a practical alternative to voxelwise NLLS for stable HP ^13^C kinetic mapping.

## Funding

This work was supported by the American Cancer Society (DICRIDG‐21‐074‐01‐DICRIDG); National Institutes of Health (1U54CA295337‐01).

## Conflicts of Interest

The authors declare no conflicts of interest.

## Supporting information


**Table S1:** Conditioning of the gamma‐variate NLLS inverse problem.
**Table S2:** Key median absolute covariance correlations among fitted parameters.
**Table S3:** Quantitative in vivo map‐summary metrics for the TRAMP mouse tumor VFA dataset.
**Table S4:** Quantitative in vivo map‐summary metrics for the rat kidney 15°/15° CFA dataset.
**Figure S1:** Practical parameter coupling in the gamma‐variate NLLS formulation.
**Figure S2A:** Agreement between **true** and **predicted** kinetic parameters for a two‐compartment HP‐^13^C model using (A) non‐linear least squares (NLLS) and (B) a hybrid multi‐head neural network. Each point is one simulated voxel (*n* = 100 time‐series pairs). Blue dashed line: identity y=x. Reported metrics are $R^{2}$ (with bootstrap SD), regression slope/intercept, and RMSE. Parameter units: $k_{\mathrm{PL}},k_{\mathrm{VE}}$ in s^−1^; $v_{B}$ unitless fraction. Simulations used a 25‐timepoint constant flip angle schedule ($\theta_{P}/\theta_{L}=15^{{}^{\circ}}/15^{{}^{\circ}}$). Axes are identically windowed across methods for each parameter.
**Figure S2B:** Selected voxel‐level fits to (A) **pyruvate** and (B) **lactate** time courses generated with a 25‐timepoint constant flip angle schedule ($\theta_{P}/\theta_{L}=15^{{}^{\circ}}/15^{{}^{\circ}}$, TR = 2 s); Black dots: observed data; **solid blue**: NN fit; **red dashed**: NLLS fit. Panels report true parameters and method estimates $\left(k_{\mathrm{PL}},k_{\mathrm{VE}}\;\left[s^{-1}\right],v_{B}\right)$ signal peak, noise standard deviation and SNR. Identical axis ranges are used within each metabolite to enable visual comparison.
**Figure S3A:** Agreement between **true** and **predicted** kinetic parameters for a two‐compartment HP‐^13^C model using (A) non‐linear least squares (NLLS) and (B) a hybrid multi‐head neural network. Each point is one simulated voxel (*n* = 100 time‐series pairs). Blue dashed line: identity y=x. Reported metrics are $R^{2}$ (with bootstrap SD), regression slope/intercept, and RMSE. Parameter units: $k_{\mathrm{PL}},k_{\mathrm{VE}}$ in s^−1^; $v_{B}$ unitless fraction. Simulations used a 12‐timepoint constant flip angle schedule ($\theta_{P}/\theta_{L}=11^{{}^{\circ}}/80^{{}^{\circ}}$, TR = 5 s). Axes are identically windowed across methods for each parameter.
**Figure S3B:** Selected voxel‐level fits to (A) **pyruvate** and (B) **lactate** time courses generated with a 12‐timepoint constant flip angle schedule ($\theta_{P}/\theta_{L}=11^{{}^{\circ}}/80^{{}^{\circ}}$, TR = 5 s); Black dots: observed data; **solid blue**: NN fit; **red dashed**: NLLS fit. Panels report true parameters and method estimates $\left(k_{\mathrm{PL}},k_{\mathrm{VE}}\;\left[s^{-1}\right],v_{B}\right)$, signal peak, noise standard deviation and SNR. Identical axis ranges are used within each metabolite to enable visual comparison.
**Figure S4A:** Brain HP‐^13^C MRI: anatomy, AUCs, and kinetic maps for subject 2. Top: T1‐weighted reference (same slice/registration), AUC_pyr_ and AUC_lac_ (a.u.), and unitless AUC_lac_/AUC_pyr_ ratio. Middle: voxelwise parameters from NLLS. Bottom: corresponding NN maps.
**Figure S4B:** Brain HP‐^13^C MRI: anatomy, AUCs, and kinetic maps for subject 3. Top: T1‐weighted reference (same slice/registration), AUC_pyr_ and AUC_lac_ (a.u.), and unitless AUC_lac_/AUC_pyr_ ratio. Middle: voxelwise parameters from NLLS. Bottom: corresponding NN maps.
**Figure S5:** Effect on NLLS generated $k_{\mathrm{PL}}$ parameter maps of increasing the allowed upper parameter bound $k_{\mathrm{PL},\max}$ (Column 4, Rows 1–3). Row 4 show the NN parameter map for comparison.

## Data Availability

The Python implementation, trained neural‐network weights, and preclinical data used for reproducing the analyses are currently publicly available at: https://github.com/kofideh/HP13CNNModel.
