# Supplementary material for: Deep Learning Improves Robustness of Voxelwise Kinetic Modeling for Hyperpolarized Carbon‐13 MRI
Source: Magn Reson Med. 2026 Aug 4;96(6):2857–69. doi: 10.1002/mrm.70549 (PMC13619905; doi:10.1002/mrm.70549)
Supplement: Supplementary file 1 [file MRM-96-2857-s001.docx]

**Supplementary Note 1. Practical identifiability of the gamma-variate NLLS formulation**

To assess the practical identifiability of the gamma-variate nonlinear least-squares (NLLS) formulation, we computed a finite-difference sensitivity matrix for the full fitted parameter vector

$$\boldsymbol{\theta}=\left[ k_{\mathrm{PL}},k_{\mathrm{VE}},v_{B},A,\alpha,\beta,t_{0} \right]$$

(1)

For each acquisition protocol, synthetic pyruvate/lactate time courses were generated using the same two-compartment model and protocol-specific timing/flip-angle schedule used in the main analysis. The gamma-variate NLLS model was fit to each time course, and the local sensitivity matrix was computed around the fitted solution using the same weighted residual vector minimized by NLLS:

$$\boldsymbol{r(\theta)}=\left[ \frac{S_{\mathrm{pyr}}^{\mathrm{pred}}(t_{i};\boldsymbol{\theta})-S_{\mathrm{pyr}}^{\mathrm{obs}}(t_{i})}{\sigma_{\mathrm{pyr}}},\frac{S_{\mathrm{lac}}^{\mathrm{pred}}(t_{i};\boldsymbol{\theta})-S_{\mathrm{lac}}^{\mathrm{obs}}(t_{i})}{\sigma_{\mathrm{lac}}} \right]_{i=1}^{T}$$

(2)

The finite-difference Jacobian was defined as

$$J_{ij}=\frac{\partial r_{i}}{\partial\theta_{j}}$$

(3)

where $r_{i}$ is the weighted residual vector used in Eq. 2, i.e., concatenated pyruvate and lactate residuals after scaling by $\sigma_{pyr}$ and $\sigma_{lac}$.

To summarize parameter coupling, we computed the column-normalized sensitivity-cosine matrix and the approximate covariance-correlation matrix derived from ($\left( J^{T}J \right)^{-1}$), using a pseudoinverse when necessary. The condition number and singular-value spectrum of the relative-sensitivity Jacobian were also evaluated. These metrics quantify local practical identifiability rather than global structural identifiability.

| **Protocol** | **Time points** | **Median condition number** | **Median minimum singular value** | **Max median (sensitivity cosine)** | **Max median (covariance corr)** |
| --- | --- | --- | --- | --- | --- |
| TRAMP VFA | 16 | 568 [348–986] | 0.00813 [0.00528–0.0143] | 0.980 | 0.997 |
| CFA 15°/15° | 25 | 653 [394–1.64e3] | 0.00733 [0.00306–0.012] | 0.979 | 0.998 |
| Human 11°/80° | 12 | 8.34e7 [5.76e3–2.36e12] | 2.93e-8 [2.32e-10–5.57e-4] | 1.000 | 1.000 |

**Supplementary Table S1. Conditioning of the gamma-variate NLLS inverse problem.** Summary of local practical identifiability metrics for the seven-parameter gamma-variate NLLS fit ([$k_{\mathrm{PL}},k_{\mathrm{VE}},v_{B},A,\alpha,\beta,t_{0}$]) across the three acquisition protocols. For each protocol, a finite-difference Jacobian was computed from the weighted pyruvate/lactate residual vector at the fitted solution. The table reports the median condition number, median minimum singular value, maximum median absolute sensitivity cosine, and maximum median absolute covariance correlation across simulated time courses. Larger condition numbers and smaller minimum singular values indicate poorer numerical conditioning, while values of sensitivity cosine or covariance correlation near 1 indicate strong local parameter coupling. The human 11° pyruvate/80° lactate protocol showed the poorest conditioning, consistent with reduced practical identifiability of the gamma-variate NLLS fit under short acquisitions with rapid lactate depletion. Values are reported as median[interquartile range] across simulated time courses. VFA = variable flip angle; CFA = constant flip angle.

| **Pair** | **TRAMP VFA (corr)** | **CFA15 (corr)** | **Human 11°/80° (corr)** |
| --- | --- | --- | --- |
| $k_{\mathrm{VE}}-A$ | 0.992 | 0.989 | 0.998 |
| $v_{B}-A$ | 0.962 | 0.931 | 0.955 |
| $k_{\mathrm{VE}}-v_{B}$ | 0.915 | 0.879 | 0.956 |
| $k_{\mathrm{VE}}-\alpha$ | 0.816 | 0.782 | 0.981 |
| $k_{\mathrm{VE}}-\beta$ | 0.878 | 0.853 | 0.984 |
| $k_{\mathrm{VE}}-t_{0}$ | 0.734 | 0.706 | 0.932 |

**Supplementary Table S2. Key median absolute covariance correlations among fitted parameters.**

Although the finite-difference Jacobian was locally full rank for all three protocols, the inverse problem was poorly conditioned. For the VFA protocol, the median condition number was 568 [348–986], and the median absolute covariance correlations were high for $k_{VE}$–$A$ (0.992), $v_{B}$–$A$ (0.962), and $k_{VE}$–$v_{B}$ (0.915). The human 11°/80° protocol was substantially more ill-conditioned, with median condition number $8.34\times{10}^{7}$. These results indicate strong practical coupling among delivery/exchange, vascular fraction, and AIF parameters, explaining why NLLS can produce visually acceptable pyruvate/lactate curve fits while yielding unstable or boundary-dominated estimates of $k_{\mathrm{VE}}$ and $v_{B}$. The neural network does not remove this fundamental information limitation, but acts as a regularized inverse estimator constrained by the physiologic training distribution.

**Supplementary Figure S1. Practical parameter coupling in the gamma-variate NLLS formulation.** Median absolute covariance-correlation matrices for the seven fitted NLLS parameters ($\left[ k_{\mathrm{PL}},k_{\mathrm{VE}},v_{B},A,\alpha,\beta,t_{0} \right]$) under the VFA TRAMP protocol (left), 15°/15° CFA protocol (middle), and 11° pyruvate/80° lactate human brain protocol (right). Values were computed from the finite-difference sensitivity matrix of the weighted pyruvate/lactate residual vector, with covariance approximated from $\left( J^{T}J \right)^{-1}$). Values near 1 indicate strong local parameter coupling. Across protocols, $k_{\mathrm{VE}},v_{B}$, and the AIF amplitude/shape parameters show substantial covariance coupling, particularly ($k_{\mathrm{VE}}$–$A$, $v_{B}$–$A$, and $k_{\mathrm{VE}}$–$v_{B}$). Coupling is most pronounced for the 11°/80° protocol, consistent with poorer practical identifiability of the gamma-variate NLLS fit under short, lactate-depleting acquisitions.

|  | NLLS | NN |
| --- | --- | --- |
| k_PL_ vs $\mathrm{AUC}_{Lac/Pyr}$ (ρ) | 0.47675 | 0.853941 |
| v_B_ vs $\mathrm{AUC}_{\mathrm{Pyr}}$ (ρ) | -0.15149 | 0.776336 |
| k_PL_ (near boundary %) | 53.28597 | 1.065719 |
| v_B_ (near boundary %) | 29.12966 | 0.35524 |

**Supplementary Table S3**. **Quantitative in vivo map-summary metrics for the TRAMP mouse tumor VFA dataset.** Spearman rank correlations ($\rho$) were computed within the foreground mask between $k_{PL}$ and the Lac/Pyr AUC ratio, and between $v_{B}$ and pyruvate AUC. The last two rows report the percentage of foreground-mask voxels lying within a distance equal to 1% of the full, method-specific admissible parameter range from either bound. For parameter $x$ with allowed range $\left[ x_{\min} , x_{\max} \right]$, a voxel was classified as near-boundary when $x\leq x_{\min}+0.01(x_{\max}-x_{\min})$or $x\geq x_{\max}-0.01(x_{\max}-x_{\min})$. These percentages quantify accumulation near the prespecified NLLS fitting or NN output bounds, not proximity to the empirical map minimum or maximum.

|  | NLLS | NN |
| --- | --- | --- |
| k_PL_ vs $\mathrm{AUC}_{Lac/Pyr}$ (ρ) | 0.97 | 0.90 |
| v_B_ vs $\mathrm{AUC}_{\mathrm{Pyr}}$ (ρ) | -0.0013 | 0.57 |
| k_PL_ within 1% of parameter bounds (%) | 3.07 | 0 |
| v_B_ within 1% of parameter bounds (%) | 93.3 | 2.45 |

**Supplementary Table S4. Quantitative in vivo map-summary metrics for the rat kidney 15°/15° CFA dataset.** Spearman rank correlations ($\rho$) were computed within the foreground mask between $k_{PL}$ and the Lac/Pyr AUC ratio, and between $v_{B}$ and pyruvate AUC. The last two rows report the percentage of foreground-mask voxels lying within a distance equal to 1% of the full, method-specific admissible parameter range from either bound. For parameter $x$ with allowed range $\left[ x_{\min} , x_{\max} \right]$, a voxel was classified as near-boundary when $x\leq x_{\min}+0.01(x_{\max}-x_{\min})$ or $x\geq x_{\max}-0.01(x_{\max}-x_{\min})$. These correspond to the NLLS fitting bounds or the NN output bounds, respectively, and not to the empirical minimum or maximum observed in a displayed map.

**Figure S2A.** Agreement between **true** and **predicted** kinetic parameters for a two-compartment HP-${}^{13}$C model using **(A)** non-linear least squares (NLLS) and **(B)** a hybrid multi-head neural network. Each point is one simulated voxel (n = 100 time-series pairs). Blue dashed line: identity $y=x$. Reported metrics are $R^{2}$ (with bootstrap SD), regression slope/intercept, and RMSE. Parameter units: $k_{\mathrm{PL}},k_{\mathrm{VE}}$ in s${}^{-1}$; $v_{B}$ unitless fraction. Simulations used a 25-timepoint constant flip angle schedule ($\theta_{P}/\theta_{L}=15^{\circ}/15^{\circ}$). Axes are identically windowed across methods for each parameter.

**Figure S2B.** Selected voxel-level fits to **(A) pyruvate** and **(B) lactate** time courses generated with a 25-timepoint constant flip angle schedule ($\theta_{P}/\theta_{L}=15^{\circ}/15^{\circ}$, TR = 2 s); Black dots: observed data; **solid blue**: NN fit; **red dashed**: NLLS fit. Panels report true parameters and method estimates $\left( k_{\mathrm{PL}},k_{\mathrm{VE}} \left[ s^{-1} \right],v_{B} \right)$ signal peak, noise standard deviation and SNR. Identical axis ranges are used within each metabolite to enable visual comparison.

**Figure S3A.** Agreement between **true** and **predicted** kinetic parameters for a two-compartment HP-${}^{13}$C model using **(A)** non-linear least squares (NLLS) and **(B)** a hybrid multi-head neural network. Each point is one simulated voxel (n = 100 time-series pairs). Blue dashed line: identity $y=x$. Reported metrics are $R^{2}$ (with bootstrap SD), regression slope/intercept, and RMSE. Parameter units: $k_{\mathrm{PL}},k_{\mathrm{VE}}$ in s${}^{-1}$; $v_{B}$ unitless fraction. Simulations used a 12-timepoint constant flip angle schedule ($\theta_{P}/\theta_{L}=11^{\circ}/80^{\circ}$, TR = 5 s). Axes are identically windowed across methods for each parameter.

**Figure S3B.** Selected voxel-level fits to **(A) pyruvate** and **(B) lactate** time courses generated with a 12-timepoint constant flip angle schedule ($\theta_{P}/\theta_{L}=11^{\circ}/80^{\circ}$, TR = 5 s); Black dots: observed data; **solid blue**: NN fit; **red dashed**: NLLS fit. Panels report true parameters and method estimates $\left( k_{\mathrm{PL}},k_{\mathrm{VE}} \left[ s^{-1} \right],v_{B} \right)$, signal peak, noise standard deviation and SNR. Identical axis ranges are used within each metabolite to enable visual comparison.

**Figure S4A.** Brain HP-${}^{13}$C MRI: anatomy, AUCs, and kinetic maps for subject 2**.** Top: T1-weighted reference (same slice/registration), AUC${}_{\text{pyr}}$ and AUC${}_{\text{lac}}$ (a.u.), and unitless AUC${}_{\text{lac}}$/AUC${}_{\text{pyr}}$ ratio. Middle: voxelwise parameters from NLLS. Bottom: corresponding NN maps.

**Figure S4B.** Brain HP-${}^{13}$C MRI: anatomy, AUCs, and kinetic maps for subject 3**.** Top: T1-weighted reference (same slice/registration), AUC${}_{\text{pyr}}$ and AUC${}_{\text{lac}}$ (a.u.), and unitless AUC${}_{\text{lac}}$/AUC${}_{\text{pyr}}$ ratio. Middle: voxelwise parameters from NLLS. Bottom: corresponding NN maps.

| Upper bound, $k_{PL,max}$(s^-1^) | $k_{PL}^{NLLS}\geq0.99k_{PL,max}$ | NLLS to NN spatial rank concordance (*Spearman* $\rho$ ) | Scale factor, c (*R^2^)*, where  $\left( k_{PL}^{\mathrm{NN}}=c\text{ }k_{PL}^{\mathrm{NLLS}} \right)$ |
| --- | --- | --- | --- |
| 0.06 | 99.4% | -0.12 | 0.65 (-2.19) |
| 0.2 | 98.6% | 0.18 | 0.20 (-0.32) |
| 1 | 77.6% | 0.38 | 0.04 (-5.97) |
| 10 | 36.4% | 0.22 | 0.0047 (-121) |

**Supplementary Table S5.** Quantitative summary metrics for $k_{PL}^{NLLS}$ maps when the allowed upper parameter bound is increased for Column 1 shows the percentage of foreground voxels with $k_{PL}^{\mathrm{NLLS}}\geq0.99\text{ }k_{PL,\max}$. This directly measures whether boundary hitting decreases as the bound is raised. Column 2 shows the foreground-voxel Spearman correlation between: $k_{PL}^{\mathrm{NLLS}}\text{ and }k_{PL}^{\mathrm{NN}}.$ Spearman correlation is unaffected by a simple positive rescaling, therefore, a genuinely scaled version of the NN map should have a high $\rho$, ideally approaching 1. Column 3. shows scale-only rescaling where the value c is fit for scale-only model: $k_{PL}^{\mathrm{NN}}=c\text{ }k_{PL}^{\mathrm{NLLS}}$.

**Supplementary Figure S5**: Effect on NLLS generated $k_{PL}$parameter maps of increasing the allowed upper parameter bound $k_{PL,max}$ (Column 4, Rows 1 to 3). Row 4 show the NN parameter map for comparison.
